# Emergence of synfire chains with triphasic spike-time-dependent plasticity

**DOI:** 10.1186/1471-2202-12-S1-P41

**Published:** 2011-07-18

**Authors:** Amelia Waddington, Peter A Appleby, Marc deKamps, Netta Cohen

**Affiliations:** 1 School of Computing, University of Leeds, Leeds, West Yorkshire, LS2 9JT, UK; 2 Institute of Systems and Membrane Biology, University of Leeds, Leeds, West Yorkshire, LS2 9JT, UK

## 

Precisely timed and sequential activity patterns are commonplace throughout the nervous system and thought to play important roles in neural processing. The generation of such precise sequences of spikes has been conjectured to rely on the existence of so-called synfire chains. Synfire chains are effectively feed-forward structures composed of multiple layers in which the activity flows from the input, sequentially through the layers, with each repetition of the input producing the same precise firing pattern [1]. Accumulating electrophysiological evidence *in vitro* and *in vivo*[2,3] suggests that the observed sequential patterns are generated by chain like structures. The possible importance of such structures has led many to ask by what neuronal, synaptic and network mechanisms synfire chains develop?

It is by now well established (e.g. [4]) that synfire chains can be grown using conventional (anti-symmetric) spike-timing-dependent plasticity (STDP) rules in which apparently causal firing patterns lead to the potentiation of the corresponding synapse whereas apparently anti-causal firing patterns lead to its depression. In all these studies, however, the STDP rule was complemented by additional topological constraints that served to limit the number of synaptic partners a neuron can have.

Here, we show that by incorporating a different class of STDP rules, it is possible to grow synfire chains in the absence of any topological constraints. Specifically, we describe a class of triphasic STDP rules that can give rise to the growth of stable chains. The emerging chains are characterized by a range of layer widths and chain lengths, whose layer-profile can be modulated by the learning rate and where this profile appears to scale nicely with network size. Using computational modeling and a coarse grained random-walk formulation, we demonstrate the role of the STDP rule in growing, molding and stabilizing the chain. Finally, we show that such triphasic rules can be used to develop multiple chains within a network.
